# Ultralow power spin–orbit torque magnetization switching induced by a non-epitaxial topological insulator on Si substrates

**DOI:** 10.1038/s41598-020-69027-6

**Published:** 2020-07-22

**Authors:** Nguyen Huynh Duy Khang, Soichiro Nakano, Takanori Shirokura, Yasuyoshi Miyamoto, Pham Nam Hai

**Affiliations:** 1 Department of Electrical and Electronic Engineering, Tokyo Institute of Technology, 2-12-1 Ookayama, Meguro, Tokyo 152-8550 Japan; 2 Department of Physics, Ho Chi Minh City University of Education, 280 An Duong Vuong Street, District 5, Ho Chi Minh City, 738242 Vietnam; 3 Science & Technology Research Labs., NHK (Japan Broadcasting Corporation), 1-10-11 Kinuta, Setagaya, Tokyo 157-8510 Japan; 4 CREST, Japan Science and Technology Agency, 4-1-8 Honcho, Kawaguchi, Saitama 332-0012 Japan; 5 Center for Spintronics Research Network (CSRN), The University of Tokyo, 7-3-1 Hongo, Bunkyo, Tokyo 113-8656 Japan

**Keywords:** Materials for devices, Nanoscale materials

## Abstract

The large spin Hall effect in topological insulators (TIs) is very attractive for ultralow-power spintronic devices. However, evaluation of the spin Hall angle and spin–orbit torque (SOT) of TIs is usually performed on high-quality single-crystalline TI thin films grown on dedicated III-V semiconductor substrates. Here, we report on room-temperature ultralow power SOT magnetization switching of a ferrimagnetic layer by non-epitaxial BiSb TI thin films deposited on Si/SiO_2_ substrates. We show that non-epitaxial BiSb thin films outperform heavy metals and other epitaxial TI thin films in terms of the effective spin Hall angle and switching current density by one to nearly two orders of magnitude. The critical SOT switching current density in BiSb is as low as 7 × 10^4^ A/cm^2^ at room temperature. The robustness of BiSb against crystal defects demonstrate its potential applications to SOT-based spintronic devices.

## Introduction

Charge-to-spin conversion utilizing the strong spin–orbit coupling (SOC) in non-magnetic materials has become a very attractive concept with possible applications to various spintronic devices, such as spin–orbit torque (SOT) magnetoresistive random access memories (MRAM)^1^, race-track memories^2^, and spin torque nano-oscillators^3,4^. These SOT-based spintronic devices are superior to their spin-transfer torque (STT)-based counterparts in terms of driving current, speed, and long-term durability^5,6^. In SOT-based devices, a perpendicular pure spin current density *J*_s_ is generated by an in-plane charge current density *J*_e_ in the non-magnetic layer through the spin Hall effect (SHE)^7–9^, whose charge-to-spin conversion efficiency is characterized by the spin Hall angle *θ*_SH_ = (2e/ℏ) *J*_s_/*J*_e_. Thus, finding spin Hall materials with large *θ*_SH_ and high electrical conductivity is crucial for SOT applications, and there have been huge efforts so far to achieve that goal. In the well-studied heavy metals (HMs) such as Pt^10–13^, Ta^14^, and W^15–17^, *θ*_SH_ is of the order of ~ 0.1, and the typical critical switching current density *J*_c_ in bilayers of heavy metals/ferromagnet with perpendicular magnetic anisotropy is typically of the order of 10^7^ A/cm^2^ for continuous direct currents (DC) and 10^8^ A/cm^2^ for nano-second (ns) pulse currents. The large switching current density requires large driving transistors, whose size limits the bit density of SOT-MRAM. Meanwhile, large *θ*_SH_ (> 1) have been observed in topological insulators (TIs)^18,19^ thanks to their strong SOC and Dirac-point-driven singularity of the Berry phase on their topologically protected surface states^20^. Thus, significant reduction of the driving current density from 10^7^–10^8^ A/cm^2^ to 10^5^–10^6^ A/cm^2^ can be expected for SOT-based devices^21–25^, particularly in SOT-MRAM whose their large writing current density is the major obstacle for reducing the writing power consumption and increasing the bit density. However, evaluation of *θ*_SH_ and SOT switching by TIs is usually performed on single-crystalline TI thin films deposited on dedicated III-V semiconductor substrates, which is not suitable for mass production and integration with Si electronics. In realistic SOT applications, the TI thin films have to be deposited directly on insulating layers in the back-end-of-line such as thermally oxidized Si, or on top of Co-based alloys or superlattices, where single crystallinity cannot be expected. Thus, it is crucial to investigate the SOT performance of non-epitaxial TI thin films on Si/SiO_2_ wafers. Recently, there are attempts to deposit and evaluate the performance of non-epitaxial Bi_*x*_Se_1-*x*_ and Bi_*x*_Te_1-*x*_ thin films by sputtering on Si/SiO_2_ substrates, which show promising results^26,27^. In particular, Bi_*x*_Se_1-*x*_ shows a very large *θ*_SH_ = 8.7–18.6 but with the expense of reduced electrical conductivity (~ 7.8 × 10^3^ Ω^−1^ m^−1^) comparing with epitaxial Bi_2_Se_3_ (~ 5.7 × 10^4^ Ω^−1^ m^−1^). When in contact with a metallic ferromagnetic layer (6 × 10^5^ Ω^−1^ m^−1^ for CoFeB), the small electrical conductivity is a big disadvantage since most of the current will flow into the metallic ferromagnetic layer and does not contribute to generation of the pure spin current. In addition, Se is a highly evaporative and toxic element, making it challenging for process integration with Si electronics. Therefore, further TI option should be explored.

Among various material candidates, BiSb, the first discovered three-dimensional TI^28–30^, stands out as a practical TI for SOT-based devices because it shows both high electrical conductivity (*σ* ~ 2.5 × 10^5^ Ω^−1^ m^−1^)^31^ and a giant spin Hall angle (*θ*_SH_ = 52 for BiSb(012) surface)^32^. Indeed, we have demonstrated ultralow power SOT magnetization switching in epitaxial BiSb(012)/MnGa bilayers grown on GaAs(001) substrates by molecular beam epitaxy (MBE). Despite the large perpendicular anisotropy field of 40–50 kOe and the large coercivity of 1.6 kOe of the MnGa layer, the SOT critical switching current density in the BiSb(012)/MnGa bilayer is as low as 1.5 × 10^6^ A/cm^2^ at room temperature. In addition, a giant unidirectional spin Hall magnetoresistance of 1.1%, which is three orders of magnitude larger than in those in metallic bilayers, has been observed in a BiSb/GaMnAs bilayer^33^.

In this work, to explore BiSb topological insulator’s potential for realistic SOT spintronic devices and integration with Si electronics, we investigate the SOT performance of non-epitaxial BiSb thin films using CoTb ferrimagnetic layers^34,35^ deposited on Si/SiO_2_ substrates. We show that non-epitaxial BiSb thin films deposited by either MBE or sputtering outperform heavy metals and other epitaxial TIs by one to nearly two orders of magnitude in terms of the effective spin Hall angle and switching current density. We demonstrate SOT magnetization switching of the CoTb layer with a threshold current density as low as 7 × 10^4^ A/cm^2^ for DC currents, and 2.2 × 10^6^ A/cm^2^ for 10 ns pulse currents. The robustness of the spin Hall effect in BiSb demonstrates its potential applications to SOT-based spintronic devices.

## Results

### SOT performance of non-epitaxial BiSb thin films deposited by MBE

**Figure 1:**
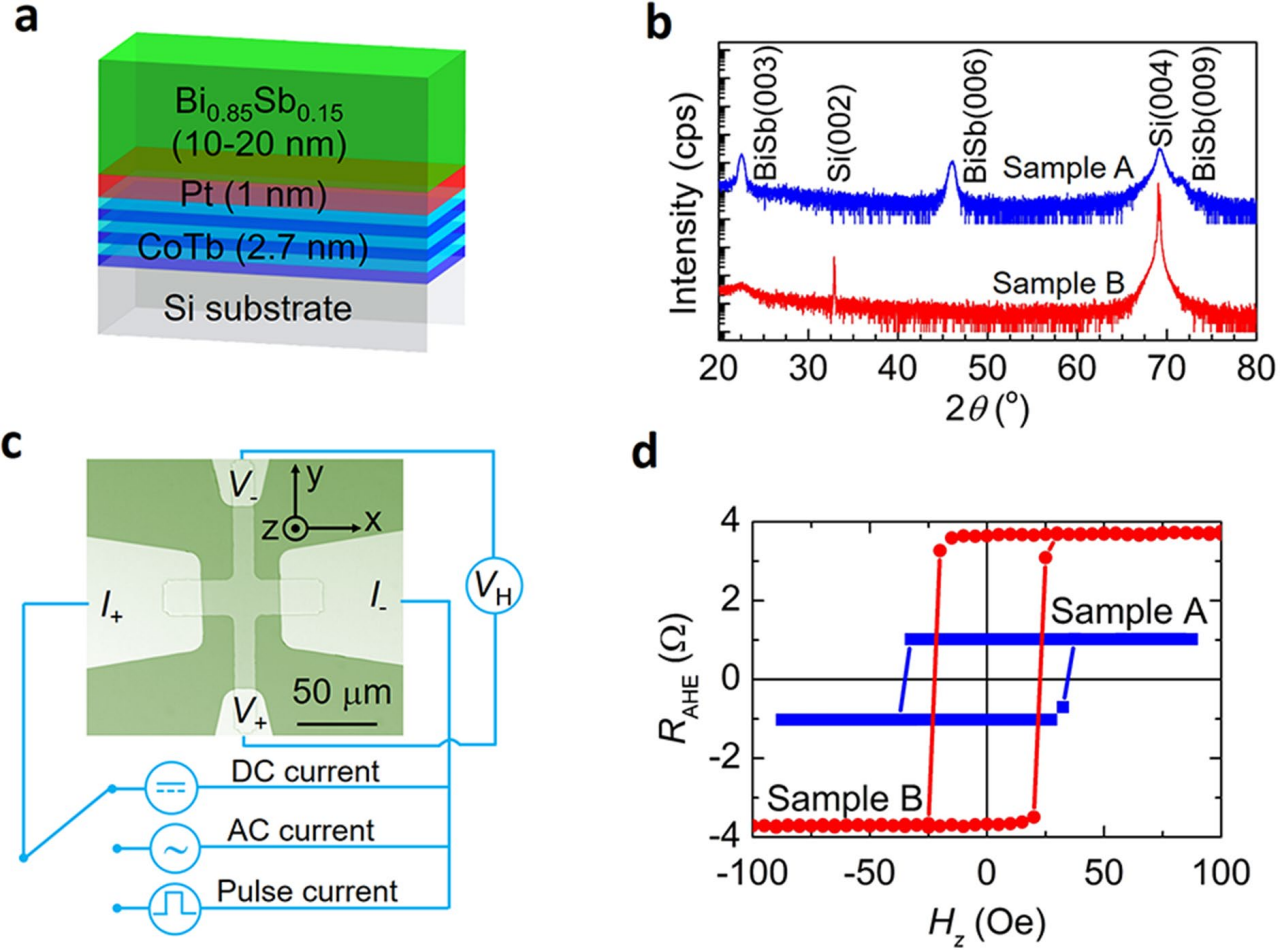
CoTb(2.7)/Pt(1)/Bi_0.85_Sb_0.15_(10–20) stack structure. (**a**) Schematic structure of the CoTb(2.7)/Pt(1)/Bi_0.85_Sb_0.15_(10–20) stack. (**b**) XRD spectra of CoTb(2.7)/Pt(1)/Bi_0.85_Sb_0.15_(20) (sample A) and CoTb(2.7)/Pt(1)/Bi_0.85_Sb_0.15_(10) (sample B) stack. **(c)** Optical image of a CoTb(2.7)/Pt(1)/Bi_0.85_Sb_0.15_(20) Hall bar device and measurement configuration. (**d**) Anomalous Hall resistance of a 25 μm-wide Hall bar of sample A (blue) and sample B (red).

We deposited stacking structures of Si/SiO_2_ substrate/CoTb(2.7)/Pt(1)/Bi_0.85_Sb_0.15_(10–20) as illustrated in Fig. 1a (the numbers in parentheses represent layer thickness in nanometer). The CoTb(2.7)/Pt(1) stack was first deposited by the ion beam sputtering method (see Method). The CoTb layer has a saturation magnetization *M*_S_ of 180 emu/cc and a perpendicular anisotropy field *H*_u_ of 1.6 kOe (see Supplementary Information Section 1). Here, we chose CoTb as the magnetic layer because there are rich benchmarking data for various CoTb/heavy metal and CoTb/TI bilayers^22^. The stack was exposed to air and transferred to an MBE chamber for deposition of the top Bi_0.85_Sb_0.15_ layer at room temperature without any cleaning or thermal treatment of the metallic layers. To study the role of the topological surface states, we prepare two samples with different Bi_0.85_Sb_0.15_ layer thickness: sample A with a 20 nm Bi_0.85_Sb_0.15_ layer and sample B with a 10 nm Bi_0.85_Sb_0.15_ layer. Figure 1b shows the X-ray diffraction (XRD) patterns of the final stacks of sample A and B. We observed weak peaks of the BiSb(001) phase comparing with those grown on dedicated GaAs(111)A substrates^29^, indicating the poorer crystal quality of the BiSb layers. In fact, we could not observe clear reflection high energy electron diffraction patterns of these layers during MBE growth. As a result, the conductivity of the deposited non-epitaxial BiSb layers is not as high as that of epitaxial BiSb thin films grown on GaAs(111)A substrates. By applying a parallel resistor model, we estimate that the electrical conductivity of the BiSb layer *σ*_BiSb_ in sample A is 9.1 × 10^4^ Ω^−1^ m^−1^, which is several times lower than that of epitaxial BiSb thin films (~ 2.5 × 10^5^ Ω^−1^ m^−1^). In sample B, *σ*_BiSb_ is even smaller (*σ*_BiSb_ = 2.7 × 10^4^ Ω^−1^ m^−1^) due to larger amount of crystal defects for the thinner film. Thus, the BiSb thin films studied here represent the worst case situation with bad crystal quality and low electrical conductivity. The ratio between the current density flowing in the BiSb layer *J*^BiSb^ and the total current density *J* in the whole stack is *J*^BiSb^/*J* = 0.7 in sample A and *J*^BiSb^/*J* = 0.3 in sample B. The samples were patterned into 25 μm-wide Hall bars by optical lithography and Ar ion milling. Hall bars fabricated from the CoTb(2.7)/Pt(1) stack without BiSb were also used as references for extraction of the electrical conductivity of the BiSb layers and investigation of artifacts related to the cap Pt(1) layer. Figure 1c shows an optical image of a 25 μm-wide Hall bar of sample A and the measurement configuration. In our experiments, the SOT magnetization switching was performed by sweeping an in-plane external magnetic field *H*_*x*_ under a constant DC current, or sweeping a DC/pulse current under a constant *H*_*x*_. For quantitative evaluation of the effective spin Hall angle in the BiSb layers, we employed the second harmonic Hall measurement with an AC current while sweeping *H*_*x*_^19,24,36^. Figure 1d shows the Hall resistance of a 25 μm-wide Hall bar of sample A and B under a perpendicular magnetic field *H*_*z*_, which show clear perpendicular magnetic anisotropy of the CoTb layer. The larger anomalous Hall resistance in sample B is consistent with the lower *σ*_BiSb_ of its 10 nm-thick BiSb top layer, since more current flows into the CoTb layer in sample B than in sample A.

**Figure 2:**
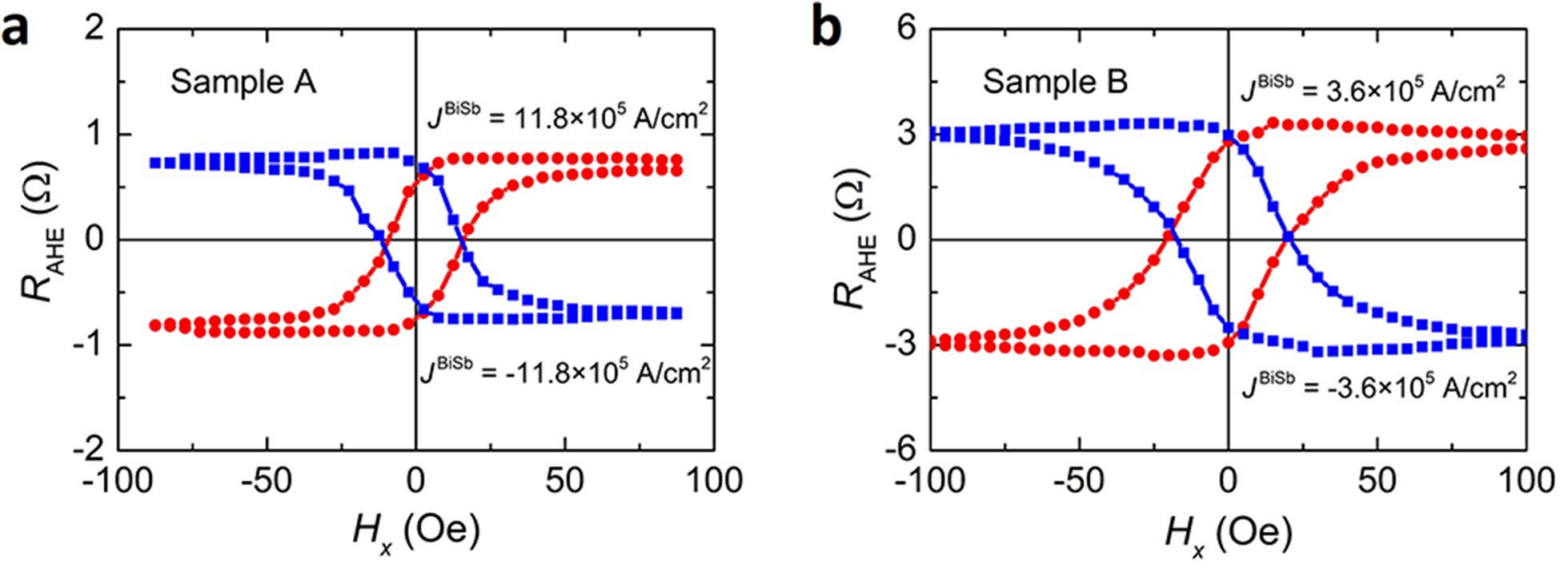
SOT magnetization switching by sweeping an in-plane magnetic field. Anomalous Hall resistance *R*_AHE_ measured as a function of an in-plane external magnetic field swept along the *x* axis for (**a**) *J*_BiSb_ =  ± 11.8 × 10^5^ A/cm^2^ in sample A, and (**b**) *J*_BiSb_ =  ± 3.6 × 10^5^ A/cm^2^ in sample B.

Figure 2a and b show the SOT switching when sweeping an in-plane external magnetic field under a DC current density of *J*^BiSb^ =  ± 11.8 × 10^5^ A/cm^2^ (*J* =  ± 1.7 × 10^6^ A/cm^2^) in sample A and *J*^BiSb^ =  ± 3.6 × 10^5^ A/cm^2^ (*J* =  ± 1.2 × 10^6^ A/cm^2^) in sample B, respectively. The magnetization of the CoTb layer in these samples was first saturated by applying a large external magnetic field *H*_*z*_ perpendicular to the film plane. Then, an in-plane external magnetic field *H*_*x*_ was swept under a positive (negative) current density. The red (blue) lines represent the perpendicular magnetization *M*_*z*_ measured under the positive (negative) current density. Clear magnetization switching was observed in both samples, whose switching direction was reversed between positive and negative current, consistent with SOT switching. Note that the corresponding *J*^BiSb^ = 3.6 × 10^5^ A/cm^2^ in sample B is nearly three times smaller than that in sample A (*J*^BiSb^ = 11.8 × 10^5^ A/cm^2^).

**Figure 3:**
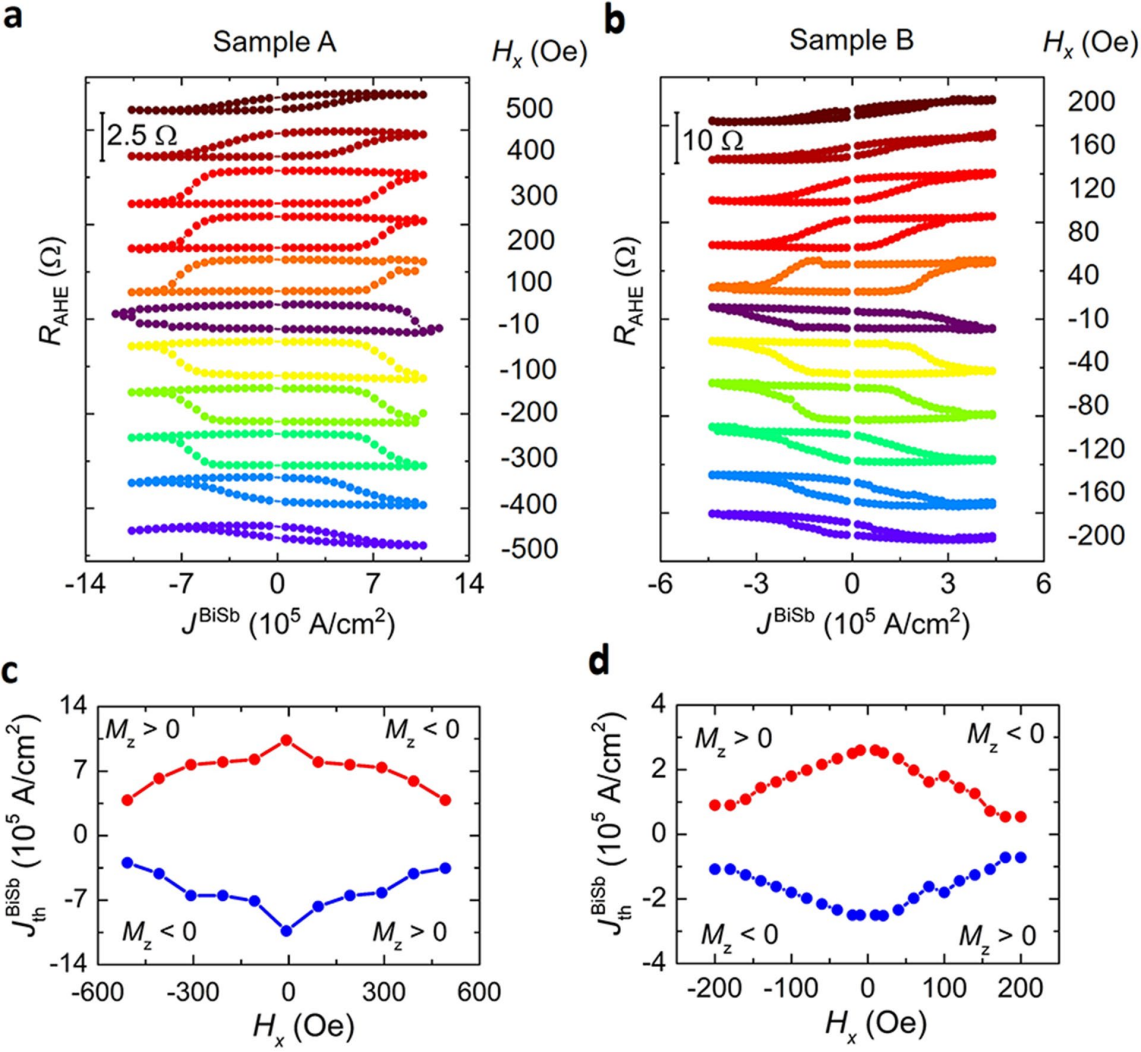
SOT magnetization switching by sweeping a DC current. Anomalous Hall resistance *R*_AHE_ measured as a function of the current density *J*_BiSb_ measured at different in-plane external magnetic field *H*_*x*_ for (**a**) sample A and (**b**) sample B. Threshold switching current density $J_{{{\text{th}}}}^{{{\text{BiSb}}}}$ as a function of *H*_*x*_ for (**c**) sample A and (**d**) sample B.

**Figure 4:**
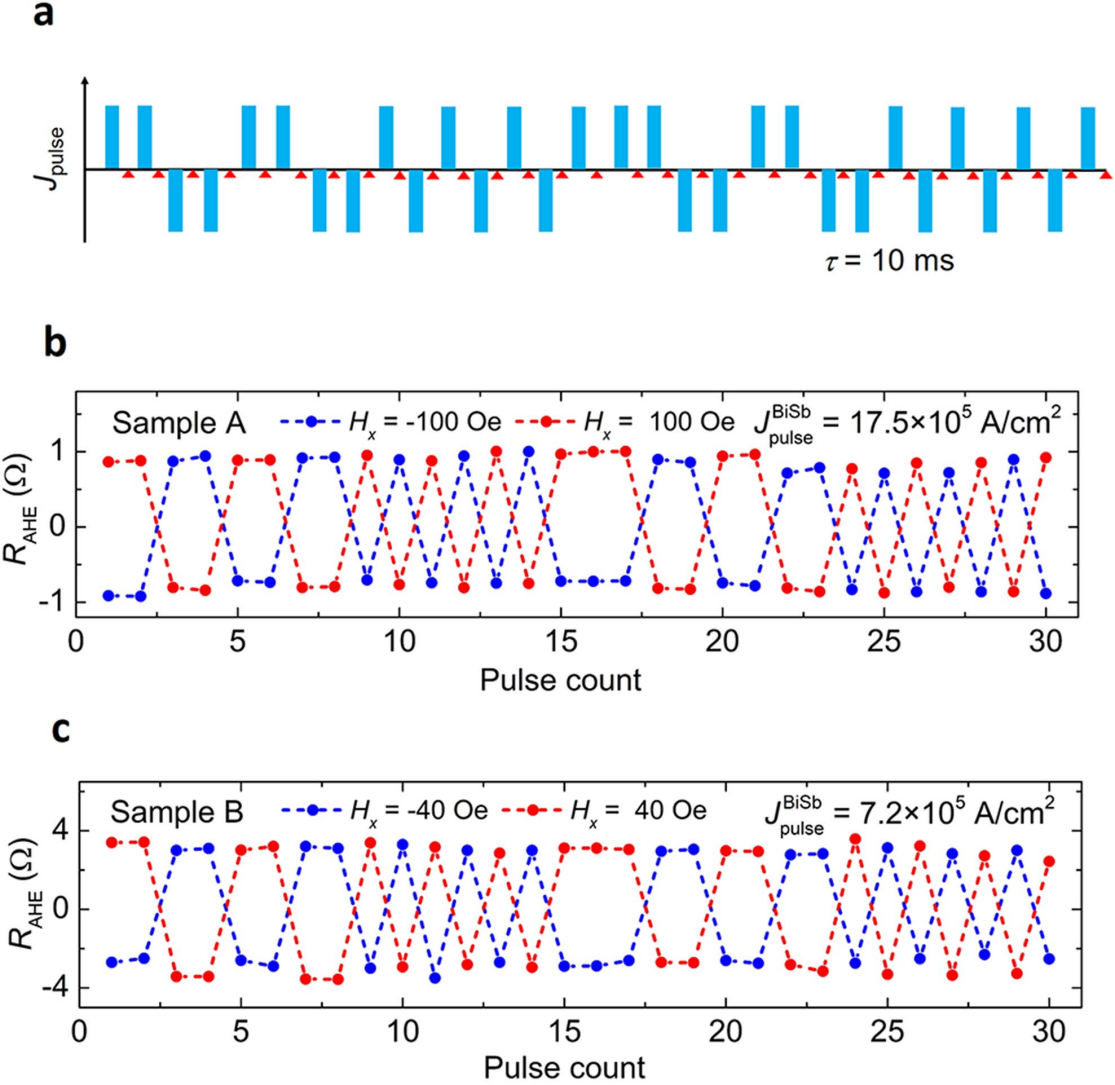
Pulse current-induced SOT magnetization switching. **(a)** Applied pulse sequence for repeating SOT magnetization switching by 10 ms pulse currents. The red triangles indicate read out of the Hall resistance after each pulse. **(b)** Pulse current-induced SOT switching of sample A at $J_{{{\text{pulse}}}}^{{{\text{BiSb}}}}$ =  ± 11.7 × 10^5^ A/cm^2^ under *H*_*x*_ =  ± 100 Oe and **(c)** sample B at $J_{{{\text{pulse}}}}^{{{\text{BiSb}}}}$ =  ± 7 × 10^5^ A/cm^2^ under *H*_*x*_ =  ± 40 Oe.

Figure 3a and b show the current-induced SOT magnetization switching of the CoTb layer under various *H*_*x*_ for sample A and sample B, respectively. The Hall resistance *R*_AHE_ was measured by sweeping a DC current under different constant *H*_*x*_ up to ± 500 Oe in sample A and ± 200 Oe in sample B. SOT magnetization switching of the CoTb layer was consistently confirmed even at the very small *H*_*x*_ = -10 Oe. The amplitude of the *R*_AHE_ curves is consistent with those observed in Fig. 2, indicating full magnetization switching. Here, the SOT effect from the Pt(1) cap layer is negligible because there is no magnetization switching observed in the reference CoTb(2.7)/Pt(1) samples (see Supplementary Information Section 1). Figure 3c and d show the threshold switching current density $J_{{{\text{th}}}}^{{{\text{BiSb}}}}$ as a function of *H*_*x*_. Here, $J_{{{\text{th}}}}^{{{\text{BiSb}}}}$ is defined at which the Hall resistance changes sign. In sample A, $J_{{{\text{th}}}}^{{{\text{BiSb}}}}$ at *H*_*x*_ =  + 500 Oe is as low as 3.5 × 10^5^ A/cm^2^, which is one to two orders of magnitude smaller than $J_{{{\text{th}}}}^{{{\text{Ta}}}}$ ~ 8 × 10^6^ A/cm^2^ at *H*_*x*_ = 500 Oe in a Ta/CoTb (1.7) bilayer and $J_{{{\text{th}}}}^{{{\text{Pt}}}}$ ~ 40 × 10^6^ A/cm^2^ at *H*_*x*_ = 1,700 Oe in a Pt/CoTb(2.1) bilayer, respectively^22^. This value is also smaller than $J_{{{\text{th}}}}^{{{\text{Bi}}_{{2}} {\text{Se}}_{{3}} }}$ = 3 × 10^6^ A/cm^2^ at *H*_*x*_ = 1,000 Oe observed in an epitaxial Bi_2_Se_3_(7.4)/CoTb(4.6) bilayer^22^. In sample B, $J_{{{\text{th}}}}^{{{\text{BiSb}}}}$ is further reduced to 7 × 10^4^ A/cm^2^ at *H*_*x*_ =  + 200 Oe, demonstrating that non-epitaxial BiSb maintains its performance as an effective spin Hall material for SOT applications. Figure 4 demonstrates repeating SOT magnetization switching by 10 ms pulse currents. We applied a random sequence of pulse currents (blue bars in Fig. 4a), after which the Hall resistance was measured (red triangles). The SOT switching with $J_{{{\text{Pulse}}}}^{{{\text{BiSb}}}}$ =  ± 17.5 × 10^5^ A/cm^2^ under *H*_*x*_ =  ± 100 Oe in sample A, and $J_{{{\text{Pulse}}}}^{{{\text{BiSb}}}}$ =  ± 7.2 × 10^5^ A/cm^2^ under *H*_*x*_ =  ± 40 Oe in sample B are shown in Fig. 4b and c, respectively. Repeatable full switching of the Hall resistance *R*_AHE_ whose sign depends on the pulse direction and *H*_*x*_ polarity was firmly demonstrated.

**Figure 5:**
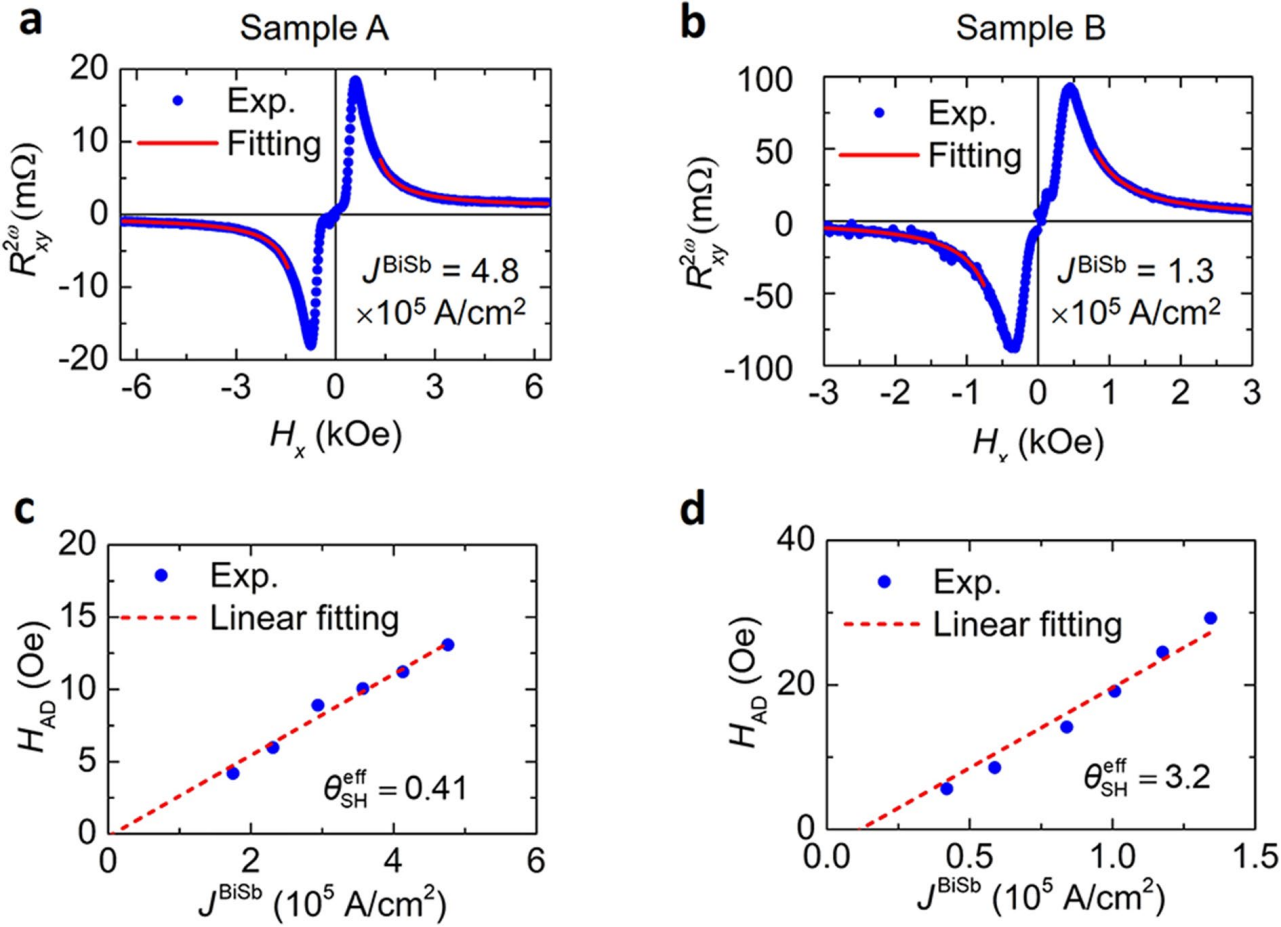
Evaluation of the effective spin Hall angle by harmonic Hall measurements. Representative second harmonic Hall resistance as a function of *H*_*x*_ at (**a**) *J*^BiSb^ = 4.8 × 10^5^ A/cm^2^ in sample A and (**b**) *J*^BiSb^ = 1.3 × 10^5^ A/cm^2^ in sample B. (**c**, **d**) Antidamping-like SOT field *H*_AD_ as a function of *J*^BiSb^ in sample A and sample B, respectively.

We further investigate the effective spin Hall angle ($\theta_{{{\text{SH}}}}^{{{\text{eff}}}}$) of the BiSb layers by employing the second Harmonic Hall measurement technique. An alternating current (AC) *J* = *J*_0_sin*ωt* (*ω*/2π = 259.68 Hz) was applied along the *x*-axis, while an in-plane external magnetic field *H*_*x*_ was swept. Under the large in-plane external magnetic field, the CoTb layers are tilted toward the *H*_*x*_ direction and are modulated by AC effective SOT magnetic fields generated by the SHE in the BiSb layers. Because of the high external magnetic field, the CoTb layers become a single domain, and the second harmonic Hall resistance $R_{xy}^{2\omega }$ measured as function of *H*_*x*_ can be expressed as $R_{xy}^{2\omega } = \frac{{R_{{{\text{AHE}}}} }}{{2}}\frac{{H_{{{\text{AD}}}} }}{{{|}H_{x} {| - }H_{{\text{u}}} }} + R_{{{\text{PNE}}}} \frac{{H_{{{\text{FL}}}} }}{{{|}H_{x} {|}}} + R_{{{\text{ANE}} + {\text{SSE}}}} \frac{{H_{x} }}{{{|}H_{x} {|}}}$, where *H*_AD_ and *H*_FL_ are the antidamping-like and field-like effective field, and *R*_AHE_ and *R*_PNE_ are the anomalous and planar Hall resistance, respectively. *R*_ANE+SSE_ is the contribution from the anomalous Nernst (ANE) and spin Seebeck (SSE) effects. The merit of the high-field second harmonic measurement is that it can distinguish the intrinsic SOT contribution from those of the thermal effects *R*_ANE+SSE_. From the *H*_AD_ − *J*^BiSb^ slope, we can calculate $\theta_{{{\text{SH}}}}^{{{\text{eff}}}} = \frac{2e}{\hbar }M_{{{\text{CoTb}}}} t_{{{\text{CoTb}}}} \frac{{\partial H_{{{\text{AD}}}} }}{{\partial J^{{{\text{BiSb}}}} }}$, where *e* is the electron charge, $\hbar$ is the reduced Plank constant, *M*_CoTb_ = 180 emu/cc and *t*_CoTb_ = 2.7 nm are the saturation magnetization and thickness of the CoTb layer. Note that in the fitting, we use the values *R*_AHE_ measured by an AC current at the frequency of 2*ω* (0.81 Ω in sample A and 2.29 Ω in sample B), which are smaller than the DC values. Figure 5a and b show the representative second harmonic Hall measurement (blue circles) and fitting results (red lines) for sample A and sample B, respectively. Figure 5c and d show the estimated antidamping-like field *H*_AD_ as a function of *J*^BiSb^ in sample A and sample B. We obtained $\theta_{{{\text{SH}}}}^{{{\text{eff}}}}$ = 0.41 for sample A and $\theta_{{{\text{SH}}}}^{{{\text{eff}}}}$ = 3.2 for sample B, the latter is much larger than that of Ta ($\theta_{{{\text{SH}}}}^{{{\text{eff}}}}$ ~ -0.03), Pt ($\theta_{{{\text{SH}}}}^{{{\text{eff}}}}$ ~ 0.017), and even larger than those of other epitaxial TI layers such as Bi_2_Se_3_ ($\theta_{{{\text{SH}}}}^{{{\text{eff}}}}$ ~ 0.16), and epitaxial (Bi,Sb)_2_Te_3_ ($\theta_{{{\text{SH}}}}^{{{\text{eff}}}}$ ~ 0.4) in junctions with CoTb^22^. The enhanced $\theta_{{{\text{SH}}}}^{{{\text{eff}}}}$ in sample B compared with that of sample A can be explained by the difference in the surface current distribution inside the BiSb layer. In both samples, most of the current in the BiSb layer flows into its surface states rather than its bulk states (see Supplementary Information Section 2). However, only the current flowing into the lower surface states, which is in contact with the underneath CoTb/Pt, contributes to SOT, while the current flowing into the upper surface states does not. In the 20 nm-thick BiSb layer of sample A, the crystal quality of the upper interface is much better than that of the lower interface, as evidenced by the much improved electrical conductivity of the 20 nm-thick BiSb layer (9.1 × 10^4^ Ω^−1^ m^−1^) comparing with that of the 10 nm-thick BiSb layer (2.7 × 10^4^ Ω^−1^ m^−1^). That means the upper interface of the 20 nm-thick BiSb is much more conductive than its lower interface, thus little current flows into the lower surface states, resulting in the reduced $\theta_{{{\text{SH}}}}^{{{\text{eff}}}}$ = 0.41 in sample A. Meanwhile, more current flows into the lower surface states in the 10 nm-thick BiSb layer of sample B with no highly conductive upper surface states, yielding a much higher $\theta_{{{\text{SH}}}}^{{{\text{eff}}}}$ = 3.2. We also measured $\theta_{{{\text{SH}}}}^{{{\text{eff}}}}$ in the sample B at different temperatures, and found that $\theta_{{{\text{SH}}}}^{{{\text{eff}}}}$ increases in correlation with more surface conduction as temperature decreases (see Supplementary Information Section 3). This highlights the role of surface states (especially the lower surface) of BiSb in generation of SOT.

Table 1 compares the effective spin Hall angle $\theta_{{{\text{SH}}}}^{{{\text{eff}}}}$, the intrinsic spin Hall angle *θ*_SH_, and the threshold switching current density $J_{{{\text{th}}}}^{{{\text{SH}}}}$ in various spin Hall layers of Ta, Pt, epitaxial Bi_2_Se_3_, epitaxial (Bi,Sb)_2_Te_3_, and our non-epitaxial 10 nm-thick BiSb in junctions with CoTb^22^. In term of $\theta_{{{\text{SH}}}}^{{{\text{eff}}}}$, the non-epitaxial 10 nm-thick BiSb outperforms heavy metals and other TIs by one to nearly two orders of magnitude. However, it was shown that $\theta_{{{\text{SH}}}}^{{{\text{eff}}}}$ of heavy metals and TIs in junctions with CoTb are typically much smaller than their intrinsic *θ*_SH_, which was attributed to the low spin transmittance of the CoTb interface. For example, *θ*_SH_ of Pt is 0.08 (Ref.^37^) but its effective $\theta_{{{\text{SH}}}}^{{{\text{eff}}}}$ with CoTb is 0.017, meaning that the spin transmittance at the Pt/CoTb interface is $T_ {\text{spin}}^{{\text{Pt/CoTb}}}$ = 21%. Thus, we can expect that the intrinsic *θ*_SH_ of the non-epitaxial BiSb can be even larger. In our samples, spins from the BiSb layers have to diffuse through the 1 nm Pt layer before reaching the CoTb interface, thus $\theta_{{{\text{SH}}}}^{{{\text{eff}}}}$ = *θ*_SH_ × $T_{\text{spin}}^{{\text{Pt/CoTb}}}$ × ${\text{sech}}(t_{{{\text{Pt}}}} /\lambda_{{{\text{SF}}}}^{{{\text{Pt}}}} )$, where *t*_Pt_ = 1 nm is the Pt thickness and $\lambda_{{{\text{SF}}}}^{{{\text{Pt}}}}$ = 1.1 nm is the Pt spin diffusion length. Therefore, we estimate that *θ*_SH_ ~ 22 for the 10 nm-thick non-epitaxial BiSb layer. Comparing with *θ*_SH_ ~ 52 obtained for epitaxial BiSb(012) thin films, the estimated *θ*_SH_ ~ 22 is quite encouraging for the BiSb(001) surface states, given that there is only one Dirac point on the (001) surface comparing with four Dirac points on the (012) surface of BiSb. Our results reconfirmed the advantage of BiSb as the spin Hall layer for SOT devices.

**Table 1:** Effective spin Hall angle $\theta_{{{\text{SH}}}}^{{{\text{eff}}}}$, intrinsic spin Hall angle *θ*_SH_, and the threshold switching current density $J_{{{\text{th}}}}^{{{\text{SH}}}}$ of several heavy metals and topological insulators in junctions with CoTb.

	$\theta_{{{\text{SH}}}}^{{{\text{eff}}}}$(with CoTb)	*θ*_SH_	$J_{{{\text{th}}}}^{{{\text{SH}}}}$(10^6^ A/cm^2^) (with CoTb)
Ta	− 0.031 (ref.^22^)	− 0.15 (ref.^14^)	~ 8 (ref.^22^)
Pt	0.017 (ref.^22^)	0.08 (ref. ^37^)	~ 40 (ref.^22^)
Epitaxial Bi_2_Se_3_	0.16 (ref.^22^)	2 ~ 3.5 (ref.^18^)	2.8 (ref.^22^)
Epitaxial (Bi,Sb)_2_Te_3_	0.4 (ref.^22^)	2.5 (ref.^24^)	NA
Non-epitaxial BiSb(001)	3.2 (this work)	~ 22	0.07 (minimum)

**Figure 6:**
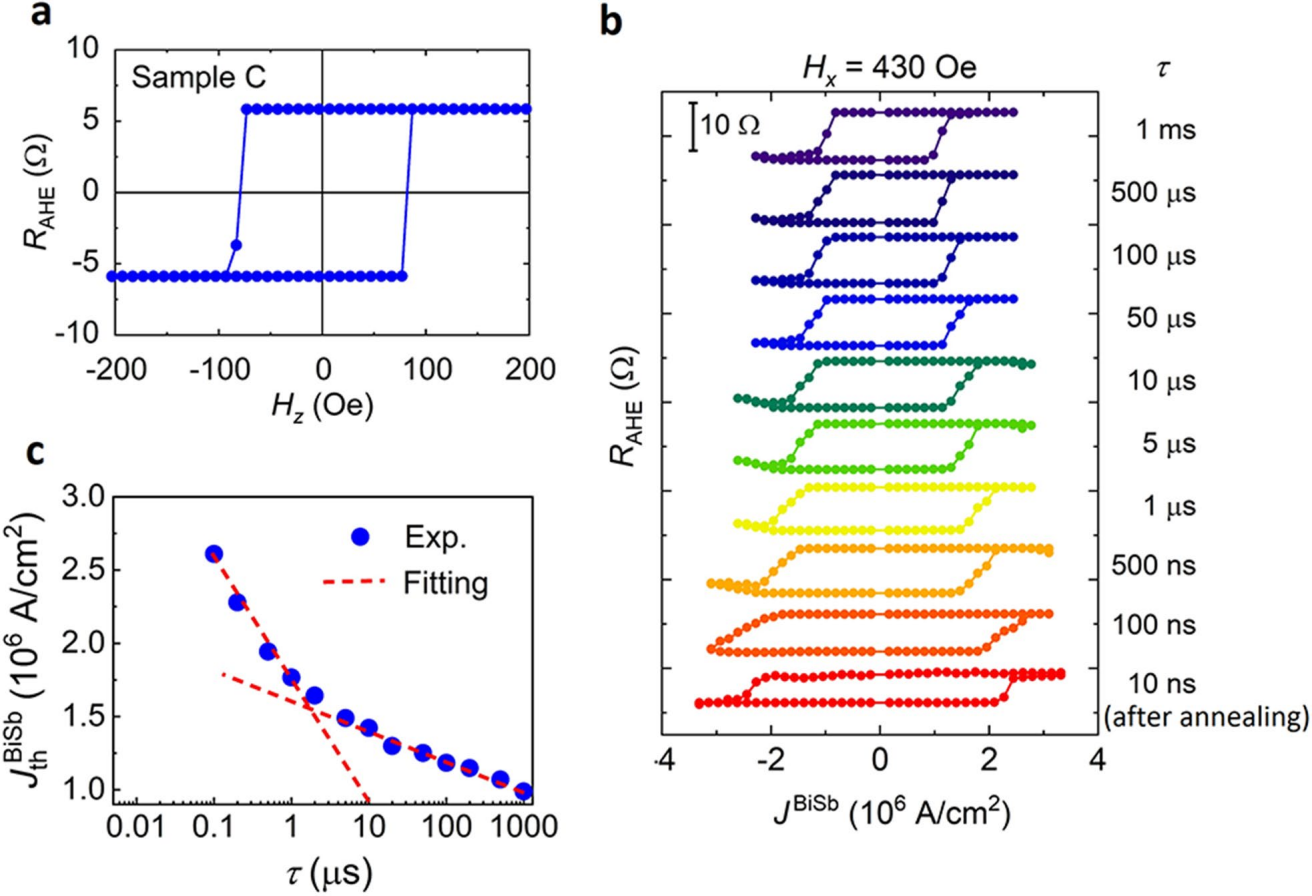
SOT magnetization switching by short pulse currents. (**a**) Anomalous Hall resistance of a 3 μm-wide Hall bar device (sample C) of the CoTb(2.7)/Pt(1)/Bi_0.85_Sb_0.15_(10) stack. (**b**) SOT magnetization switching measured at various pulse width *τ* down to 10 ns and *H*_*x*_ = 430 Oe. The switching at *τ* = 10 ns was performed after the sample was annealed at 250 °C for 30 min. (**c**) Threshold switching current density $J_{{{\text{th}}}}^{{{\text{BiSb}}}}$(before annealing) as a function of *τ*. Red dashed lines are fitting using the thermal activation model with $J_{{0}}^{{{\text{BiSb}}}}$ = 2.2 × 10^6^ A/cm^2^ and Δ = 25 for *τ* > 2 μs, and $J_{{0}}^{{{\text{BiSb}}}}$ = 4.3 × 10^6^ A/cm^2^ and Δ = 12 for *τ* < 2 μs.

To further confirm the giant spin Hall effect of the 10 nm-thick non-epitaxial BiSb layer, we investigate the SOT magnetization switching using short pulse currents with different pulse width *τ*. For this purpose, we fabricated a 3 μm-wide Hall bar with a 10 nm-thick BiSb top layer (here denoted as sample C). Figure 6a shows the anomalous Hall resistance of sample C. As shown in Fig. 6b, we achieved SOT pulse switching down to *τ* = 100 ns for this sample. We then annealed the sample C at 250 °C for 30 min to improve the crystal quality of the CoTb layer, and achieved SOT switching at *τ* = 10 ns. Figure 6c plots $J_{{{\text{th}}}}^{{{\text{BiSb}}}}$ as a function of *τ* (before annealing). One can see that $J_{{{\text{th}}}}^{{{\text{BiSb}}}}$ can be fitted by the thermal activation model $J_{{{\text{th}}}}^{{{\text{BiSb}}}}$ = $J_{{0}}^{{{\text{BiSb}}}}$_×_$\left[ {1 - \frac{1}{\Delta }\ln \left( {\frac{\tau }{{\tau_{0} }}} \right)} \right]$ with $J_{{0}}^{{{\text{BiSb}}}}$ = 2.2 × 10^6^ A/cm^2^ and Δ = 25 for *τ* > 2 μs, and $J_{{0}}^{{{\text{BiSb}}}}$ = 4.3 × 10^6^ A/cm^2^ and Δ = 12 for *τ* < 2 μs. Here, $J_{{0}}^{{{\text{BiSb}}}}$ is the threshold switching current density at 0 K, Δ is the thermal stability factor, and 1/*τ*_0_ = 1 GHz is the thermally activated switching frequency^38^. The existence of two different sets of $J_{{0}}^{{{\text{BiSb}}}}$ and Δ can be explained by the magnetic properties of CoTb. Since the sample is a μm-size Hall bar, the magnetization switching occurs not by coherent rotation of a single domain but by domain nucleation and propagation, which are affected by the local magnetic properties of CoTb. In the case of magnetization reversal by domain nucleation, Δ ≈ *π*^3^*At*_CoTb_/4*k*_B_*T* $\propto$ *T*_C_*M*_S_, where *A* is the exchange stiffness constant and *T*_C_ is the Curie temperature^39^. Meanwhile, $J_{{0}}^{{{\text{BiSb}}}}$ is proportional to the magnetic anisotropy constant *K*_u_. Because CoTb is a ferrimagnet with opposite magnetic moments for Co and Tb, its magnetic properties depend on the local composition of Co and Tb. Our CoTb layer has the nominal Tb composition of 41%. Near this Tb composition, domains with locally higher Tb composition will have lower *M*_S_, lower *T*_C_, but higher *K*_u_^40^. Thus, domains with locally higher Tb composition can be accounted for lower Δ but higher $J_{{0}}^{{{\text{BiSb}}}}$ values observed at pulse widths shorter than 1 μs.

After annealing, the composition fluctuation in CoTb is reduced. Thus, the large $$J_{{0}}^{{{\text{BiSb}}}}$$ is eliminated, and the observed $$J_{{{\text{th}}}}^{{{\text{BiSb}}}}$$ at 10 ns (2.2 × 10^6^ A/cm^2^) agrees with the small $$J_{{0}}^{{{\text{BiSb}}}}$$. Using $$J_{{0}}^{{{\text{BiSb}}}}$$$$= \frac{{2eM_{{\text{S}}} t_{{{\text{CoTb}}}} }}{{\hbar \theta_{{{\text{SH}}}}^{{{\text{eff}}}} }}\left( {\frac{{H_{{\text{u}}} }}{{2}} - \frac{{H_{x} }}{\sqrt 2 }} \right)$$^41^, we obtain $$\theta_{{{\text{SH}}}}^{{{\text{eff}}}}$$ = 3.3, which is nearly the same as that obtained from the second harmonic measurements. These results firmly confirm the giant $$\theta_{{{\text{SH}}}}^{{{\text{eff}}}}$$ in the non-epitaxial BiSb layer.

### SOT performance of non-epitaxial BiSb thin films deposited by sputtering

Although a large $\theta_{{{\text{SH}}}}^{{{\text{eff}}}}$ = 3.2 in the non-epitaxial 10 nm-thick BiSb film was observed, the crystal quality of the MBE-grown BiSb films on CoTb(2.7)/Pt(1) is not good, resulting on the reduced conductivity. In the case of MBE growth, the kinetic energy of Bi/Sb atoms is about 52 meV, which is not enough for high-quality crystallization of BiSb at room temperature, especially on the Pt layer with a dissimilar crystal structure and lattice constant. Furthermore, MBE is not suitable for mass production of SOT devices. In this section, we investigate the SOT performance of BiSb deposited by the sputtering technique, which provides more kinetic energy for Bi/Sb atoms for better crystallization and is widely used in mass production of MRAM. The drawback of the sputtering technique is that high kinetic energy Bi/Sb atoms may damage or diffuse into the interface of the underlying CoTb(2.7)/Pt(1) layers, thus reducing $\theta_{{{\text{SH}}}}^{{{\text{eff}}}}$.

**Figure 7:**
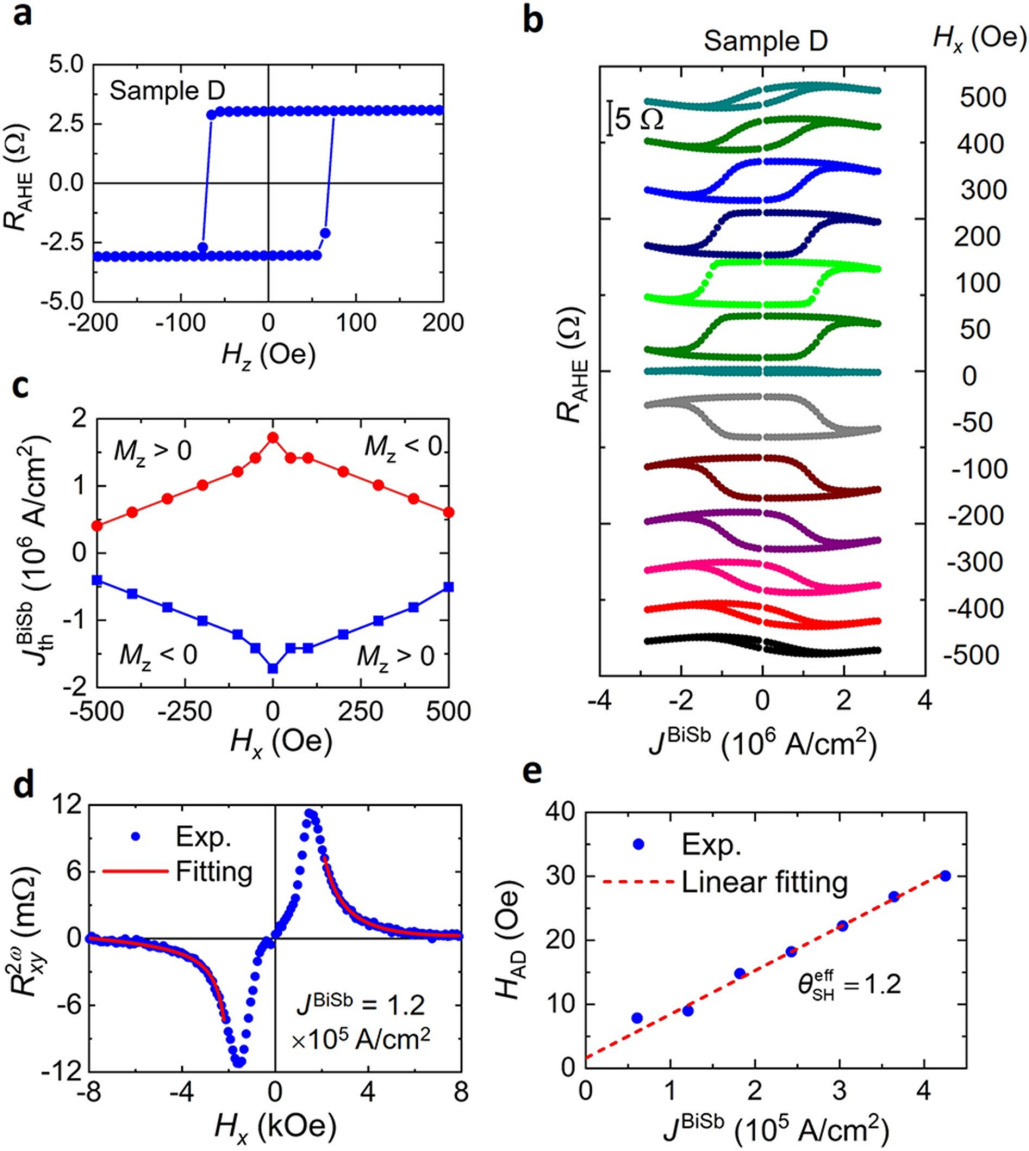
DC current-induced SOT magnetization switching and effective spin Hall angle of BiSb deposited by the sputtering technique. (**a**) Anomalous Hall resistance of a 25 μm-wide Hall bar of the CoTb(2.7)/Pt(1)/Bi_0.85_Sb_0.15_(10) stack (sample D), whose Bi_0.85_Sb_0.15_(10) was deposited by the sputtering technique. (**b**) SOT magnetization switching measured at different *H*_*x*_. (**c**) Threshold witching current density $J_{{{\text{th}}}}^{{{\text{BiSb}}}}$ as a function of *H*_*x*_. (**d**) Representative second harmonic Hall resistance as a function of *H*_*x*_ at *J*^BiSb^ = 1.2 × 10^5^ A/cm^2^. (**e**) Antidamping-like SOT field *H*_AD_ as a function *J*^BiSb^.

We deposited a 10 nm-thick Bi_0.85_Sb_0.15_ layer on top of the CoTb(2.7)/Pt(1) stacks in a multi-cathode chamber by co-sputtering Bi and Sb targets with Ar plasma at room temperature. The electrical conductivity of the sputtered BiSb layer, estimated from the parallel resistor model, is 1.1 × 10^5^ Ω^−1^ m^−1^, which is much larger than that of MBE-grown 10 nm-thick BiSb, indicating improved crystal quality thanks to higher Bi/Sb kinetic energy. The conductivity of the BiSb layer is close to those of BiSb deposited directly on sapphire substrates by sputtering^42^. Figure 7a shows the anomalous Hall resistance of a 25 μm-wide Hall bar of the CoTb(2.7)/Pt(1)/sputtered BiSb(10) stack (sample D). Figure 7b shows SOT magnetization switching in sample D by DC currents at various *H*_*x*_, while Fig. 7c plots $J_{{{\text{th}}}}^{{{\text{BiSb}}}}$ as a function of *H*_*x*_. Although $J_{{{\text{th}}}}^{{{\text{BiSb}}}}$ of sample D is higher than that of sample B, it is still as low as 4 × 10^5^ A/cm^2^ at *H*_*x*_ = 500 Oe. We then evaluate its $\theta_{{{\text{SH}}}}^{{{\text{eff}}}}$ by the second harmonic technique, whose representative $R_{xy}^{2\omega }$ − *H*_*x*_ curve and theoretical fitting are shown in Fig. 7d. Finally, from the *H*_AD_ − *J*^BiSb^ gradient, we deduced $\theta_{{{\text{SH}}}}^{{{\text{eff}}}}$ ~ 1.2 (intrinsic *θ*_SH_ ~ 8) for sample D as shown in Fig. 7e. Comparing with the sample B’s $\theta_{{{\text{SH}}}}^{{{\text{eff}}}}$ = 3.2 with the MBE-grown BiSb layer, $\theta_{{{\text{SH}}}}^{{{\text{eff}}}}$ of sample D is reduced. This can be explained by the damage or diffusion of Bi/Sb atoms into the CoTb/Pt interface due to higher impinging kinetic energy of Bi/Sb atoms. To test this hypothesis, we prepared another 10 nm-thick BiSb layer by sputtering with Kr plasma, which yields even higher kinetic energy of the Bi/Sb atoms due to heavier Kr atomic mass. The resulting BiSb layer yields a lower $\theta_{{{\text{SH}}}}^{{{\text{eff}}}}$ = 0.9, thus confirming our hypothesis that the lower $\theta_{{{\text{SH}}}}^{{{\text{eff}}}}$ of sputtered BiSb is due to the damage of the underneath CoTb/Pt interface, rather than by its own crystal quality. Our results indicate that management of the interface between BiSb and magnetic layers is an important issue for BiSb-based SOT devices.

**Figure 8:**
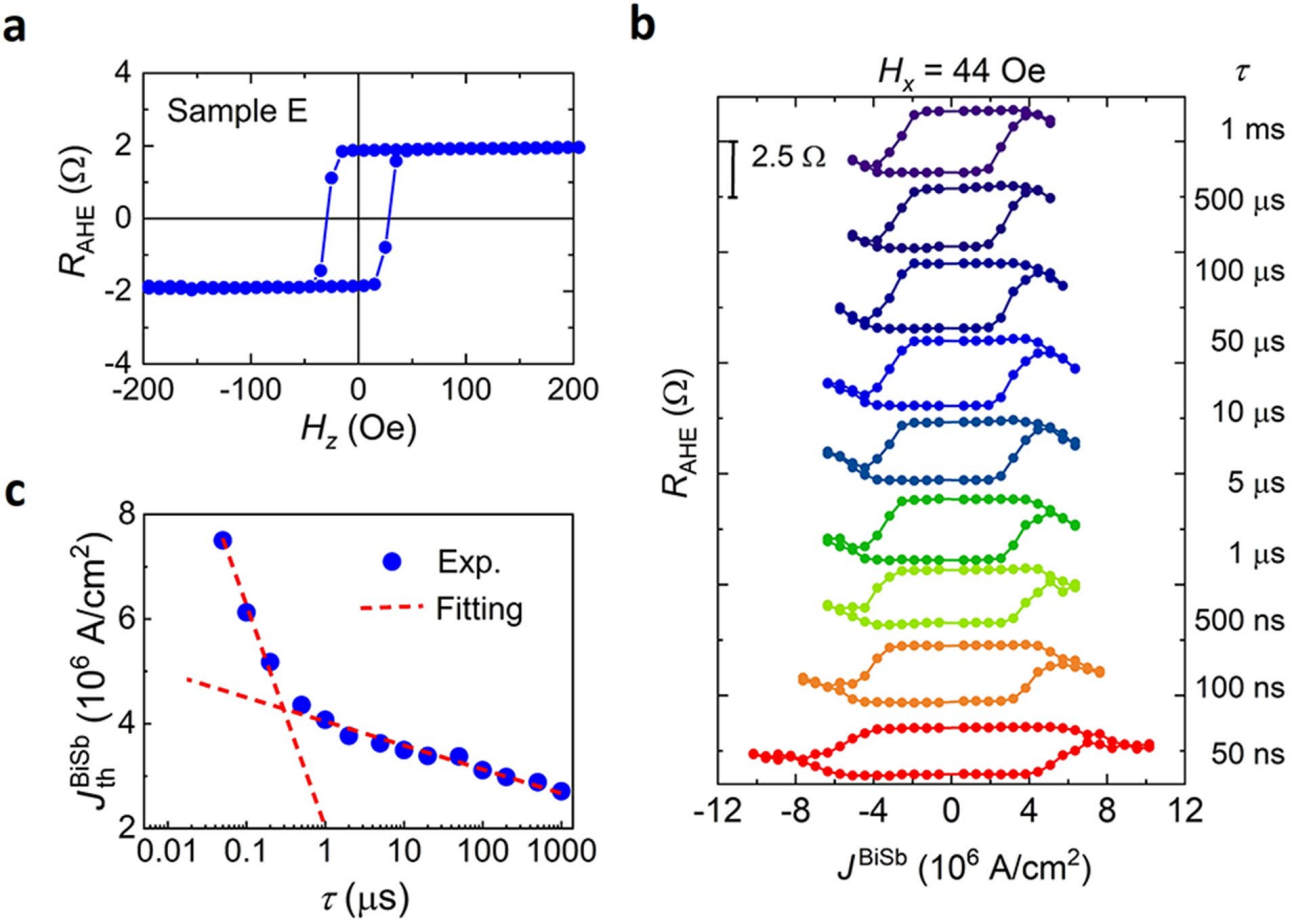
SOT magnetization switching by short pulse currents with BiSb fabricated by the sputtering technique. (**a**) Anomalous Hall resistance of a 3 μm-wide Hall bar of the CoTb(2.7)/Pt(1)/Bi_0.85_Sb_0.15_(10) stack (sample E). (**b**) Pulse-current induced SOT magnetization switching at various pulse widths *τ* down to 50 ns and *H*_*x*_ = 44 Oe. (**c**) Critical switching current density $J_{{{\text{th}}}}^{{{\text{BiSb}}}}$ as a function of *τ*. Red dashed lines are fitting using the thermal activation model with $J_{{0}}^{{{\text{BiSb}}}}$ = 5.2 × 10^6^ A/cm^2^ and Δ = 29 for *τ* > 300 ns, and $J_{{0}}^{{{\text{BiSb}}}}$ = 1.5 × 10^7^ A/cm^2^ and Δ = 8 for *τ* < 300 ns.

Finally, we performed pulse current-induced SOT switching in a 3 μm-side Hall bar of the CoTb(2.7)/Pt(1)/sputtered BiSb(10) stack (sample E). Figure 8a shows the anomalous Hall resistance of sample E, while Fig. 8b shows its SOT magnetization switching at various pulse widths *τ* down to 50 ns. Figure 8c plots $J_{{{\text{th}}}}^{{{\text{BiSb}}}}$ as a function of *τ*, which can be fitted to the thermal activation model with $J_{{0}}^{{{\text{BiSb}}}}$ = 5.2 × 10^6^ A/cm^2^ and Δ = 29 for *τ* > 300 ns, and $J_{{0}}^{{{\text{BiSb}}}}$ = 1.5 × 10^7^ A/cm^2^ and Δ = 8 for *τ* < 300 ns. The higher $J_{{0}}^{{{\text{BiSb}}}}$ in sample E compared with that of sample C agrees with the lower $\theta_{{{\text{SH}}}}^{{{\text{eff}}}}$ in sputtered BiSb compared with that grown by MBE.

## Discussion

We have demonstrated ultralow power room-temperature SOT magnetization switching induced by non-epitaxial BiSb topological insulator thin films deposited by MBE and sputtering on Si/SiO_2_ substrates. The large effective spin Hall angle was confirmed for both MBE-grown ($\theta_{{{\text{SH}}}}^{{{\text{eff}}}}$ = 3.2) and sputtered BiSb thin films ($\theta_{{{\text{SH}}}}^{{{\text{eff}}}}$ = 1.2), which are nearly two orders of magnitude larger than those of heavy metals, and even larger than those of other epitaxial TIs in junctions with CoTb, demonstrating BiSb's potential as a spin Hall material for ultralow-power SOT devices. We also show that $\theta_{{{\text{SH}}}}^{{{\text{eff}}}}$ is limited not by the quality of BiSb itself but the interface between BiSb and the underlying magnetic layers. This work helps determine the lower-bound performance of BiSb in practical conditions, and is a step closer to realization of ultralow power TI-based spintronic devices.

## Methods

### Material growth

Multilayer structures of Si/SiO_2_/CoTb(2.7)/Pt(1)/Bi_0.85_Sb_0.15_(10–20) were prepared for SOT characterization. First, the CoTb (2.7)/Pt(1) stacks were deposited by ion beam sputtering on Si/SiO_2_ substrates. The CoTb layers were formed by depositing a 0.6 nm-thick Tb and a 0.3 nm-thick Co layer alternatingly for three cycles. Then, the stacks were exposed to air and transferred to an MBE or a sputtering chamber for deposition of Bi_0.85_Sb_0.15_ (10–20 nm) layers. The Pt(1) layer protects the CoTb(2.7) layer from oxidation during air exposure.

### Device fabrication

The samples were patterned into 100 μm-long × 25 μm-wide or 18 μm-long × 3 μm-wide Hall bars by optical lithography and Ar ion milling. A 50 nm-thick Au and a 5 nm-thick Cr adhesion layer were deposited as electrodes by electron beam evaporation, which reduces the effective length of the devices to 50 μm or 9 μm.

### SOT characterization

The samples were mounted inside a cryostat equipped with an electromagnet. The cryostat was vacuumed to minimize thermal gradient across the samples during DC and harmonic measurements. The samples were in air during pulse current measurements, but were covered by a photoresist for protection. For the harmonic measurements, a NF LI5650 lock-in amplifier was employed to detect the first and the second harmonic Hall voltages under sine wave excitation generated by a Keithley 6221 AC/DC current source. For the DC current-induced SOT magnetization switching, a Keithley 2400 SourceMeter was used as the current source, and the Hall signal was measured by a Keithley 2182A NanoVoltmeter. For the pulse current-induced SOT magnetization switching, the pulses were generated by a Tektronix AFG3251C function generator, after that the Hall voltage was measured by the Keithley 2182A NanoVoltmeter under a 10 s pulse of 0.1 mA.

## Supplementary information

Supplementary file1.

## Data Availability

The data that support this study results are available from the corresponding author upon reasonable request.
