# Supplementary material for: Ultralow power spin–orbit torque magnetization switching induced by a non-epitaxial topological insulator on Si substrates
Source: Sci Rep. 2020 Jul 22;10:12185. doi: 10.1038/s41598-020-69027-6 (PMC7376042; doi:10.1038/s41598-020-69027-6)
Supplement: Supplementary file 1 — Supplementary file1. [file 41598_2020_69027_MOESM1_ESM.pdf]

Supplementary Information

Ultralow power spin-orbit torque magnetization switching induced by a non-epitaxial topological insulator on Si substrates

Nguyen Huynh Duy Khang[1,2], Soichiro Nakano[1], Takanori Shirokura[1], Yasuyoshi Miyamoto[3,5], and Pham Nam Hai[1,4,5*]

[1]Department of Electrical and Electronic Engineering, Tokyo Institute of Technology, 2-12-1 Ookayama, Meguro, Tokyo 152-8550, Japan

[2]Department of Physics, Ho Chi Minh City University of Education, 280 An Duong Vuong Street, District 5, Ho Chi Minh City 738242, Vietnam

[3]Science & Technology Research Labs, NHK (Japan Broadcasting Corporation), 1-10-11 Kinuta, Setagaya, Tokyo 157-8510, Japan

[4]Center for Spintronics Research Network (CSRN), The University of Tokyo, 7-3-1 Hongo, Bunkyo, Tokyo 113-8656, Japan

[5]CREST, Japan Science and Technology Agency, 4-1-8 Honcho, Kawaguchi, Saitama 332-0012, Japan

1. Characterization of the CoTb(2.7)/Pt(1) stack

Figure S1(a) and S1(b) show the in-plane and out-of-plane magnetization curve of the CoTb(2.7)/Pt(1) stack measured by superconducting quantum interference devices (SQUID), which show the saturation of magnetization of 180 emu/cc and the magnetic anisotropy field of 1.6 kOe for CoTb. Figure S1(c) and S1(d) show the Hall resistance of a Hall bar of the stack as the function current density under $H_x = \pm$ 200 Oe. We confirmed no SOT magnetization switching in the CoTb(2.7)/Pt(1) stack even at high $J \sim 6\times 10^6$ A/cm^2 due to the very small θ_{SH}^{eff} of the Pt(1) layer. Indeed, the second harmonic measurement showed no signal of the spin orbit torque from the Pt(1) layer.

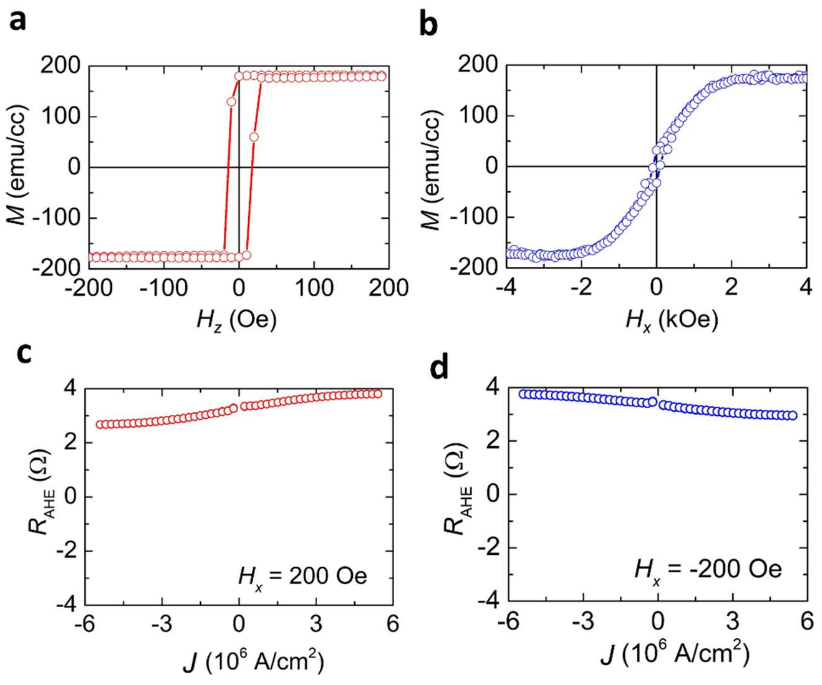

Figure S1. (a) Out-of-plane and **(b)** in-plane magnetization curve of the CoTb(2.7)/Pt(1) stack measured by superconducting quantum interference devices. **(c) (d)** Hall resistance of the stack as a function of the current density J under $H_x = \pm 200$ Oe.

2. Surface state conduction in the BiSb layers

We first determined the electrical conductivity of the BiSb layer at each temperature in sample A and sample B from the parallel resistor model of the CoTb/Pt and the BiSb layer. Then, we estimated the contribution of surface states and the bulk states to the total conductivity of the BiSb layer in sample A and sample B by using the parallel conduction model of the surface states and the bulk states. One distinct feature of BiSb from other well-known V-VI topological insulators such as Bi_2Se3, Bi_2Te_3 or Sb_2Te_3, is that its bulk is always an intrinsic semiconductor with the Femi level in the band gap. This is because Bi and Sb are in the same V-group, thus deviation of the composition or existence of anti-site defects does not result in any donors / acceptors that would generate free carriers and shift the Fermi level to the conduction band (as in the case of Bi_2Se_3 or Bi_2Te_3 due to anti-site Se/Te) or to the valence band (as in the case of Bi_2Te_3 or Sb_2Te_3 due to anti-site Bi/Sb). Therefore, the temperature dependence of the total electrical conductivity $\sigma(T)$ of BiSb is given by [42]

$$\sigma(T) = \frac{\sigma_{\mathrm{Surface}}}{t} + \sigma_{\mathrm{Bulk}} = \frac{\sigma_{\mathrm{Surface}}}{t} + \sigma_0 \exp(-\frac{E_{\mathrm{g}}}{2k_{\mathrm{B}}T}) \qquad \text{(S2.1)}$$

where $\sigma_{\mathrm{Surface}}$ is the sheet conductivity of the surface states (both upper and lower surfaces), σ_{Bulk} is the bulk conductivity, t is the thickness of BiSb, and E_{g} is the effective bandgap of BiSb including the intrinsic band gap and the extrinsic bandgap due to quantum confinement.

Figure S2(a) shows the measured temperature dependence of the normalized resistivity of the 20 nm-thick BiSb layer in sample A and the 10 nm-thick BiSb layer in sample B, as well as the theoretical fitting using equation (S2.1). For the 20 nm-thick BiSb layer, the effective bandgap is $E_{\mathrm{g}} \sim 126$ meV, which is already much larger than the intrinsic bandgap of 20 meV due to the quantum confinement effect. When the thickness is reduced to 10 nm, quantum confinement further increases the bandgap to $E_{\mathrm{g}} \sim 200$ meV. We can also determine the surface contribution to

the total conductivity $\Gamma = \frac{I_{\text{Surface}}}{I_{\text{Surface}} + I_{\text{Bulk}}} = \frac{\sigma_{\text{Surface}}}{t} \Big/ \left(\frac{\sigma_{\text{Surface}}}{t} + \sigma_{\text{Bulk}} \right)$, where I_{Surface} and I_{Bulk} are the current flowing into the surface states and the bulk states, respectively. Figure S2(b) shows Γ as a function of temperature. For the 20 nm-thick $Bi_{0.85}Sb_{0.15}$ layer, the surface state conduction accounts for 89% of the total current in the BiSb layer at room temperature. When the thickness is reduced to 10 nm, nearly all current flows into the surface of BiSb ($\Gamma \sim$ 97%) even at room temperature.

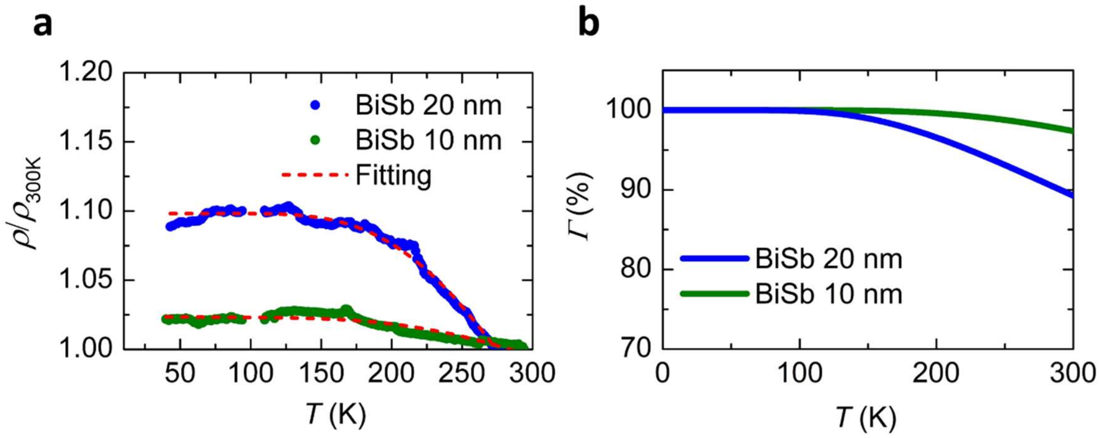

Figure S2. (a) Temperature dependence of the normalized resistivity of the 20 nm-thick $Bi_{0.85}Sb_{0.15}$ layer in sample A and the 10 nm-thick $Bi_{0.85}Sb_{0.15}$ layer in sample B. **(b)** Surface state contribution (both upper and lower surfaces) to the total conductivity Γ.

3. Dependence of the effective spin Hall angle θ_{SH}^{eff} on temperature in sample B

Figure S3(a)-S3(c) show the $H_{AD} - J^{BiSb}$ relationship measured at 320 K, 275 K, and 250 K for sample B. Figure 3(d) shows θ_{SH}^{eff} and M_{CoTb} as functions of temperature. θ_{SH}^{eff} increases in correlation with more surface conduction as temperatures decreases.

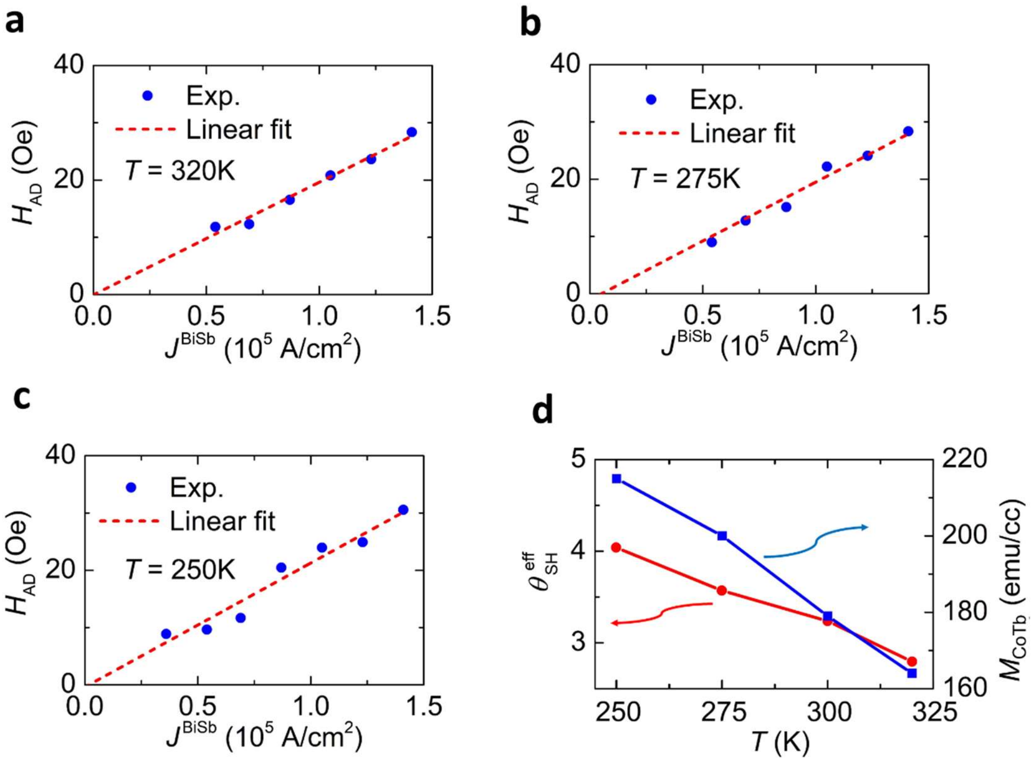

Figure S3. $H_{AD} - J^{BiSb}$ relationship in sample B measured at **(a)** $T = 320$K, **(b)** $T = 275$K, and **(c)** $T = 250$K. **(d)** θ_{SH}^{eff} (red) and magnetization M_{CoTb} (blue) as functions of temperature.
